# Nexmifa Regulates Axon Morphogenesis in Motor Neurons in Zebrafish

**DOI:** 10.3389/fnmol.2022.848257

**Published:** 2022-03-31

**Authors:** Yu-qin Zheng, Gui-hai Suo, Dong Liu, Hai-ying Li, You-jia Wu, Hong Ni

**Affiliations:** ^1^Division of Brain Science, Institute of Pediatric Research, Children’s Hospital of Soochow University, Suzhou, China; ^2^Department of Pediatrics, Affiliated Hospital of Nantong University, Nantong, China; ^3^School of Life Sciences, Nantong University, Nantong, China

**Keywords:** nexmifa, motor neuron, axon morphogenesis, efna5b, sema6ba, zebrafish

## Abstract

Nexmif is mainly expressed in the central nervous system (CNS) and plays important roles in cell migration, cell to cell and cell-matrix adhesion, and maintains normal synaptic formation and function. Nevertheless, it is unclear how nexmif is linked to motor neuron morphogenesis. Here, we provided *in situ* hybridization evidence that nexmifa (zebrafish paralog) was localized to the brain and spinal cord and acted as a vital regulator of motor neuron morphogenesis. Nexmifa deficiency in zebrafish larvae generated abnormal primary motor neuron (PMN) development, including truncated Cap axons and decreased branches in Cap axons. Importantly, RNA-sequencing showed that nexmifa-depleted zebrafish embryos caused considerable CNS related gene expression alterations. Differentially expressed genes (DEGs) were mainly involved in axon guidance and several synaptic pathways, including glutamatergic, GABAergic, dopaminergic, cholinergic, and serotonergic synapse pathways, according to Kyoto Encyclopedia of Genes and Genomes (KEGG) pathway annotation. In particular, when compared with other pathways, DEGs were highest (84) in the axon guidance pathway, according to Organismal Systems. Efna5b, bmpr2b, and sema6ba were decreased markedly in nexmifa-depleted zebrafish embryos. Moreover, both overexpression of efna5b mRNA and sema6ba mRNA could partially rescued motor neurons morphogenesis. These observations supported nexmifa as regulating axon morphogenesis of motor neurons in zebrafish. Taken together, nexmifa elicited crucial roles during motor neuron development by regulating the morphology of neuronal axons.

## Introduction

Motor neuron diseases (MNDs) are characterized by muscle weakness and/or spastic paralysis and are an etiologically heterogeneous group of disorders resulting from motor neuron degeneration (Babin et al., 2014). Thus, exploring mechanisms underpinning motor neuron development may support and advance therapeutic strategies for MND.

The zebrafish model is a highly practical *in vivo* research tool for studying developmental mechanisms, as their transparent embryos, at all developmental stages, are easy to image and manipulate (Nozawa et al., 2017). In particular, the motor neurons of the spinal cord are excellent *in vivo* systems for studying mechanisms controlling axon extension and synaptic formation (Babin et al., 2014). Growth cones at axon tips navigate using environmental cues, therefore, axons constantly follow stereotypical pathways to their targets and rarely deviate (Hilario et al., 2010). Zebrafish contain two different type of spinal motor neuron, i.e., primary motor neurons (PMNs) and secondary motor neurons (SMNs), which are based on several morphological features: soma shape, size, position, and axon diameter (Myers, 1985; Myers et al., 1986). PMNs are further divided into three groups: caudal primary motor neurons (Cap), middle primary motor neurons (Mip), and rostral primary motor neurons (Rop) in accordance with specific axonal pathways and soma positions within the spinal cord (Myers et al., 1986; Westerfield et al., 1986). Although the somata of the three identifiable PMNs are localized at different positions in the spinal cord, their axons travel to the myoseptum via a common exit point; after leaving the spinal cord, PMNs extend their axons via a common pathway to the horizontal myoseptum (Eisen et al., 1986). Finally, Cap, Mip, and Rop neurons extend their axons according to specific pathways to innervate dorsal, middle, and ventral trunk musculature, respectively (Myers, 1985; Moreno and Ribera, 2009). When compared with PMNs, SMNs are localized more ventrally in the motor column, with typically smaller somatas and thinner axons, which are born 5–6 h later than PMNs (Myers et al., 1986). Because of this unique stratification, PMNs are excellent cell systems for elucidating motor axon guidance mechanisms (Beattie, 2000; Beattie et al., 2002).

In vertebrates, motor neuron development is regulated by many genes. For example, mecp2 knockdown in zebrafish increases abnormal axonal branching of aps and decreases motor activity (Nozawa et al., 2017). lncrps25 is co-expressed with mnx1 at the spinal cord and is essential for motor neuron development, but neurons lacking lncrps25 result in Cap axon truncation and abnormal branching, however, these defects are rescued by olig2 overexpression (Gao et al., 2020). Similarly, HuD mutants exhibit decreased motor axon branches, dramatically fewer dendrites, and movement defects (Hao le et al., 2017). Deleted or overexpressed colXIX causes stumpy-like Cap axon defects (Hilario et al., 2010), whereas colXVIII knockdown causes Cap axon stalling soon after exiting the spinal cord (Schneider and Granato, 2006). Ccdc80-l1 is implicated in motor neuron axonal path-finding, however, loss-of-function does not prevent PMN formation and axon projection, but leads to PMN disorganization (Brusegan et al., 2012).

In our previous studies, we reported that insm1a, kinesin-12, and sox2 had key roles in motor neuron development (Xu et al., 2014; Gong et al., 2017, 2020). For example, the zebrafish insm1a mutant showed motor neuron loss and defects in PMN axons, including truncated length, excessive Cap branches, and disorganized distances between adjacent Caps, which were caused by the ectopic departure of motor axons from the spinal cord. In sox2 mutant zebrafish, besides truncated length and excessive Cap branches, defective PMNs also included changes in axon morphology and reductions in Mips and Rops.

Nexmif (also called KIDLIA, KIAA2022, or Xpn) is a novel gene localized to Xq13.2 (Gilbert et al., 2020). Cantagrel et al. (2004) first reported the gene in two males with intellectual disability. However, very little is known about nexmif. Previous studies reported that nexmif mRNA was strongly expressed in the cortex, hippocampus, cerebellum, and olfactory bulb (Allen Institute for Brain Science, 2004; Cantagrel et al., 2009). At the protein level, nexmif is specifically distributed in post-mitotic neuron nuclei but not in glia. Strong protein expression is also detected from the E17 developmental stage through adulthood in mice (Gilbert and Man, 2016). Thus, nexmif may have key roles in brain development.

In zebrafish, nexmif has two paralogs, nexmifa and nexmifb. Protein homology indicates 34 and 42% identity to the human protein, respectively. In humans, patients with nexmif mutations present with moderate to severe intellectual impairment, autism spectrum disorder (ASD), dystonia, intellectual disability, epilepsy, microcephaly, and facial deformities (Van Maldergem et al., 2013; de Lange et al., 2016). In animal models, nexmif was shown to participate in neurite morphological development, regulate cell migration, cell to cell and cell to matrix adhesion, and maintain normal synaptic formation and function (Ishikawa et al., 2012; Magome et al., 2013; Gilbert et al., 2020). For example, in the nexmif knockdown mouse, synapse density, spine density, and the expression of synaptic related proteins, such as AMPAR, PSD-95, and gephyrin were decreased. Also, immature spines were increased, synaptic transmission functions were defective, and mice exhibited ASD behaviors (Gilbert et al., 2020). In other work, KIAA2022 (nexmif alias) knockdown markedly suppressed neurite growth, including both dendrites and axons in cultured rat hippocampal neurons (Van Maldergem et al., 2013). Moreover, KIDLIA (nexmif alias) knockdown altered *in vivo* neuron migration, reduced dendritic growth, and disorganized apical dendrite projections in mouse layer II/III cortical neurons (Gilbert and Man, 2016). Magome et al. (2013) found that nexmif knockout inhibited cell migration by enhancing cell to cell and cell to matrix adhesion mediated by *N*-cadherin and β1- integrin in PC12 cells. However, no study has yet focused on the effects of nexmif on spinal motor neurons. Evidentially, nexmif deficiency leads to ASD behaviors, and 50–80% of patients with ASD show motor dysfunction (Kaur et al., 2018), therefore, we hypothesized nexmif exerted effects on the development of spinal motor neurons.

To verify our hypothesis, we assessed nexmifa expression using whole *in situ* hybridization (WISH) and reverse transcription-polymerase chain reaction (RT-PCR) in zebrafish. We then investigated nexmifa function during PMN morphogenesis via knockdown and knockout strategies in the Tg(mnx1:GFP)ml2 transgenic zebrafish line and investigated possible molecular mechanisms.

## Materials and Methods

### Zebrafish Lines and Breeding

Zebrafish embryos and adults were maintained in at the Zebrafish Center of Nantong University in accordance with guidelines outlined in previous studies (Xu et al., 2014; Gong et al., 2017, 2020). The transgenic zebrafish line, Tg(mnx1:GFP)ml2 and Tg(kdrl:EGFP) line have been described in the previous work (Flanagan-Steet et al., 2005; Jin et al., 2005).

### Cell Separation, RNA Isolation, Reverse Transcription, Quantitative RT-PCR and RT-PCR

At 72 h post-fertilization (hpf), we collected 300–400 Tg(mnx1:GFP) zebrafish embryos and washed them three times in phosphate-buffered saline with Tween 20 and the same again in calcium-free Ringer’s solution. Embryos were digested in 0.25% trypsin, then 10% fetal bovine serum was added to terminate the reaction. The volume was filtered through 100 and 40 μm filter membranes. Samples were then analyzed by flow cytometry (BD, Franklin Lakes, NJ, United States). Cells expressing GFP were identified as positive cells. Total RNA was extracted from zebrafish embryos and cells separated via flow cytometry by TRIzol reagent according to manufacturer’s instructions (Invitrogen, Waltham, MA, United States). Contamination was removed by DNaseI (Roche, Basel, Switzerland) and then 2 μg total RNA was reversibly transcribed using a reverse first-strand cDNA synthesis kit (Fermentas, Waltham, MA, United States) and stored at −20°C. Quantitative RT-PCR was performed using corresponding primers (Supplementary Table S1) in a 20 μL final reaction volume with 10 μL SYBR premix (Takara, Kyoto, Japan). Elongation factor 1a was used as the internal control. All samples were analyzed in triplicate. RT-PCR was performed using corresponding primers (Supplementary Table S1) in a 50 μL final reaction volume, with 25 μL 2 × Taq enzyme mix (Vazyme, Nanjing, China). After amplification, 20 μL was taken for gel electrophoresis and sequencing.

### Whole *in situ* Hybridization

A 424-base pair (bp) cDNA fragment from a wild-type embryo was amplified using nexmifa F1 and R1 primers (Supplementary Table S1). Digoxigenin (DIG)-labeled sense and antisense probes were synthesized using a linearized pGEM-Teasy vector and sub-cloned with the nexmifa fragment by *in vitro* transcription with a DIG-RNA labeling kit (Roche, Basel, Switzerland). We collected zebrafish embryos at different developmental stages (20, 48, 72, and 96 hpf), then fixed them in 4% paraformaldehyde for 2 h at room temperature or overnight at 4°C. They were then dehydrated through a series of increasing methanol concentrations, and finally stored in 100% methanol at −20°C. WISH was performed as previously described (Gong et al., 2020).

### The sgRNA/Cas9 mRNA Synthesis and Injection

Cas9 mRNA was generated by *in vitro* transcription with the linearized pXT7-Cas9 plasmid as previously described (Gong et al., 2017). sgRNAs were transcribed from the DNA templates that amplified by PCR with a pT7 plasmid as the template, a specific forward primer and a universal reverse primer (Supplementary Table S1; Gong et al., 2017, 2020). The transgenic zebrafish line Tg(mnx1:GFP)ml2 was naturally mated to obtain embryos for microinjection. Then, 1–2 cell stage zebrafish embryos were injected in a 2–3 μL solution containing 250 ng/μL Cas9 mRNA and 15 ng/μL sgRNA. At 24 hpf, embryos were randomly sampled for genomic DNA extraction according to previous methods to identify a founder. Mutant sites were verified by comparison to the wild-type unaffected sequences (chimerism). Chimeric zebrafish were mated with wild-type fish to obtain F1 fish. After examine its genotype by sequence, heterozygotic mutants were mated with *Tg(mnx1:GFP)* transgenic fish to breed the F2 generation. At last, nexmifa+/+ and nexmifa−/− littermates were obtained by F2 in-cross followed by fluorescence selection and PCR genotyping for the following experiments (Gong et al., 2017).

### Morpholino, mRNA Synthesis, and Microinjection

The nexmifa splice-blocking Morpholino (MO) and the standard control MO (Std MO) were synthesized by Gene Tools. The sequences are: 5′-AAAATGGTAGGAGTTATAAATGAGT-3′ and 5′-CCTCTTACCTCAGTTACAATTTATA-3, respectively. MOs were diluted to 0.3 mM in RNase-free water, injected into one-cell stage embryos, and then raised in E3 medium at 28.5°C to generate nexmifa knockdown embryos (morphants). To perform rescue experiments, we generated nexmifa mRNA, efna5b mRNA, and sema6ba mRNA *in vitro*. Briefly, we cloned zebrafish nexmifa, efna5b, and sema6ba separately into PCS2^+^ vectors. Next, we linearized plasmids, then *in vitro* synthesized mRNA using the mMESSAGE mMACHIN Kit (Ambion, Austin, Texas, United States) according to manufacturer’s instructions. Finally, we purified Capped mRNAs using the RNeasy Mini Kit (Qiagen, Hilden, Germany). MOs or mRNAs were injected into the yolk of one cell stage embryos using borosilicate glass capillaries (Sarasota, Florida, United States) and a PV830 pneumatic picopump (Sarasota, Florida, United States).

### The cDNA Library Preparation and RNA Sequencing

We extracted total RNA from nexmifa morphants and wild-type zebrafish at 72 hpf using TRIzol reagent (Invitrogen) and calculated RNA integrity and purity by NanoDRop 2000 (Thermo Fisher Scientific Inc., Waltham, MA, United States). Only high-quality RNA samples (OD_260/280_ = 1.8–2.2, RNA Integrity Number ≥ 8.0) were used to construct the sequencing library. We next quantified and sequenced the final cDNA libraries using the Illumina NovaSeq 6000 platform, with 2 × 150-bp pair-end reads (Illumina, San Diego, CA, United States).

### Locomotion Analysis of Zebrafish Larvae

To determine whether nexmifa deficiency impaired motility and whether this impaired motility could be rescued by overexpress the possible downstream gene, larva zebrafish at 7 days post-fertilization (dpf) in different groups were placed into 24-well-culture plates (one larva/well) and transferred to the Zebralab Video-Track system (Zebrabox, Lyon, France). The unit was equipped with a sealed opaque plastic box insulated from the environment, and an infrared filter and monochrome camera. After 30 min adaptation, larval distances and average speeds were recorded for 30 min.

### Microscopy

After anesthetizing zebrafish embryos by tricaine (Sigma, Saint Louis, Missouri, United States), they were embedded in 0.8% low melting agarose and examined using a Leica TCS-SP5 LSM confocal imaging system. Criteria for zebrafish embryos with abnormal PMNs were as follows: firstly, Caps length or the number of Caps branches per 1mm was less than 70% of the average of normal wild-type zebrafish. Secondly, PMNs abnormal in more than two hemisegment in the spinal cord in one fish. Otherwise, the embryo was normal. *In situ* hybridization images were Captured on an Olympus stereomicroscope MVX10.

### Statistical Analysis

Statistical data comparisons were performed using Student’s *t*-test or one-way analysis of variance if the data follow a normal distribution and variance between groups was uniform, otherwise, Kruskal–Wallis *H* or Mann–Whitney *U* was used. A *p* < 0.05 value was considered statistically significant. Statistical analyses were performed in SPSS 21.0 software (SPSS, Armonk, NY, United States).

## Results

### Nexmifa Is Expressed in the Spinal Cord and PMNs of Zebrafish

**FIGURE 1:**
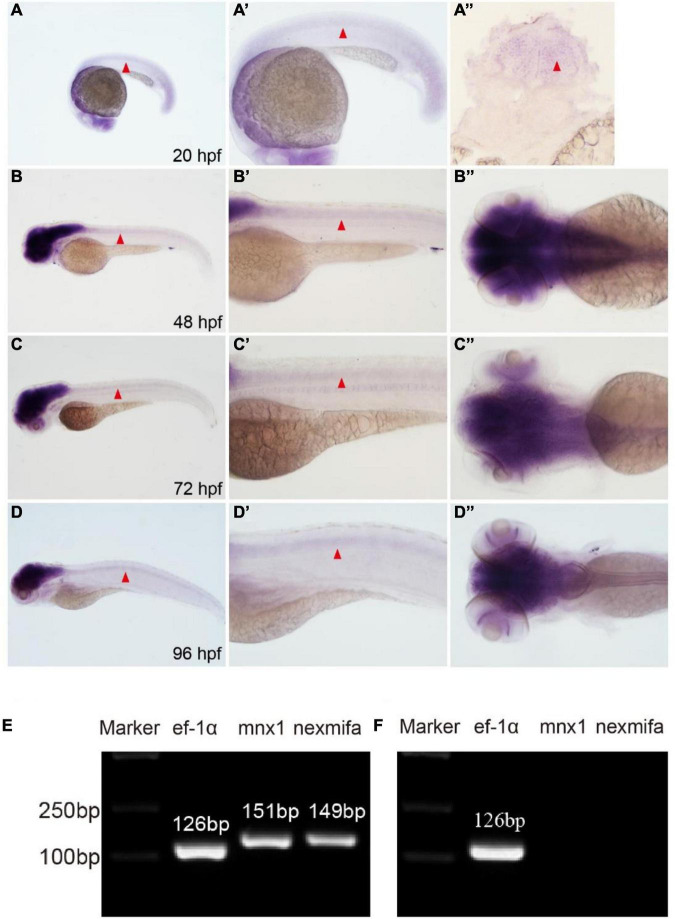
Nexmifa expression analysis in spinal cord and motor neurons. **(A)** At 20 hpf, *in situ* hybridization nexmifa signals are localized in brain and spinal cord (lateral view). **(A’)** Partially magnified image of **(A)**. **(A”)** The trunk transverse section of the embryo. **(B)** At 48 hpf, *in situ* hybridization nexmifa signals are localized in the brain and spinal cord (lateral view). **(B’)** Partially magnified image of **(B)**. **(B”)** Dorsal view of the brain. **(C)** At 72 hpf, *in situ* hybridization nexmifa signals are localized in the brain and spinal cord (lateral view). **(C’)** Partially magnified image of **(C)**. **(C”)** Dorsal view of the brain. **(D)** At 96 hpf, *in situ* hybridization nexmifa signals are localized in the brain and spinal cord (lateral view). **(D’)** Partially magnified image of **(D)**. **(D”)** Dorsal view of the brain. **(E)** RT-PCR results on mnx1-GFP sorted cells. Nexmifa is expressed in selected neuron cells from the Tg (mnx1: EGFP) line. **(F)** The result of the RT-PCR on Kdrl-EGFP sorted cells. No signals of nexmifa and mnx1 are detected in the GFP-positive cells sorted from the Tg(kdrl:EGFP) line.

To analyze nexmifa temporal and spatial expression patterns, we performed WISH using a DIG-labeled nexmifa probe at different times. Nexmifa was strongly expressed in the central nervous system (CNS), including the brain and spinal cord at 20, 48, 72, and 96 hpf. At 48 h, expression in the brain and spinal cord was the highest, but then decreased gradually (Figures 1A–D, A′–D′, A″–D″). To further assess if nexmifa was expressed in motor neurons in the spinal cord, we separated motor neurons from Tg(mnx1:GFP)ml2 cells and extracted RNA, as Tg(mnx1:GFP)ml2 motor neurons were GFP labeled. RT-PCR demonstrated that both mnx1 and nexmifa were present in selected neuron cells (Figure 1E) suggesting nexmifa was expressed in zebrafish motor neurons. Moreover, we performed RT-PCRs on nexmifa-negative tissue. The results showed that no nexmifa and mnx1 signal were detected in the GFP-positive cells sorted from the Tg(kdrl:EGFP) line (Figure 1F), in which endothelial cells were labeled with GFP.

### Nexmifa Loss Causes Motor Neurons Defects

**FIGURE 2:**
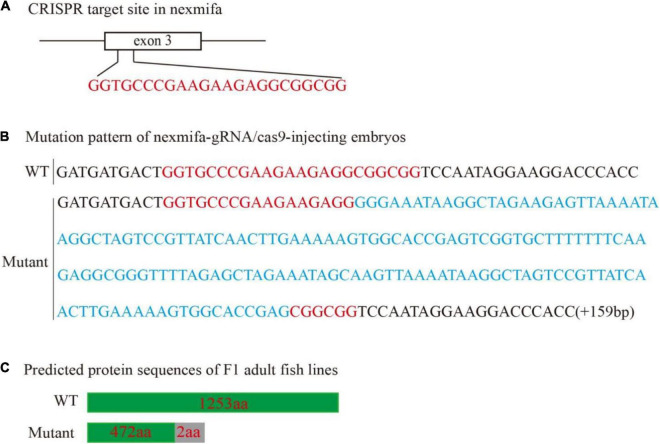
Generation of the zebrafish nexmifa mutant using the CRISPR/Cas9 system. **(A)** Schematic showing the targeting site of the sgRNA in the third exon of nexmifa. **(B)** Mutation pattern of nexmifa-gRNA/cas9-injected embryos. Red letters represent the sgRNA sequence. Blue letters represent the additional 159 bp nucleotide sequence. **(C)** Schematic showing the predicted protein encoded by the mutated allele. Frameshift mutations resulted in truncated proteins. The gray rectangle indicates the wrong coded amino acid sequences.

**FIGURE 3:**
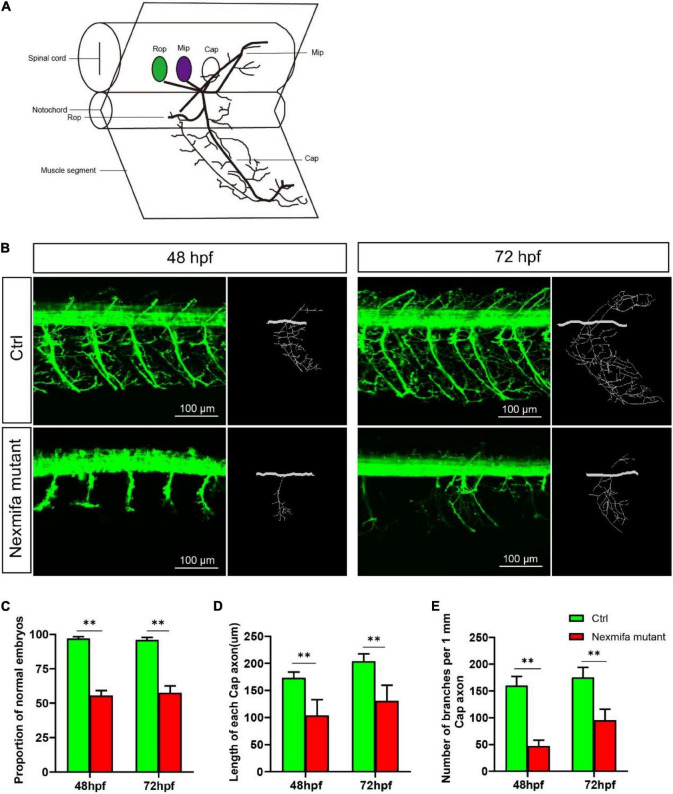
Nexmifa affects motor neuron morphogenesis in nexmifa mutant zebrafish embryos. **(A)** The schematic for three different primary motoneurons (CaP, MiP, and RoP) in fish. **(B)** Confocal imaging of primary motor neurons in control and nexmifa mutant groups at 48 and 72 hpf. Scale bar = 100 μm **(C)** The percentage of embryos with normal PMNs in control and nexmifa mutants at 48 hpf (*n* = 125 and 240, respectively) and 72 hpf (*n* = 132 and 235, respectively). **(D)** Cap length in control and nexmifa mutants at 48 hpf (*n* = 20 and 31, respectively) and 72 hpf (*n* = 15 and 28, respectively). **(E)** Number of branches per 1 mm Cap axon in control and nexmifa mutants at 48 hpf (*n* = 8 and 10, respectively) and 72 hpf (*n* = 9 and 12, respectively). Bar represent the mean ± standard deviation (SD). ^**^p < 0.01.

To explore if nexmifa regulated motor neuron morphogenesis in the spinal cord, we established a nexmifa knockout in Tg(mnx1:GFP)ml2 transgenic zebrafish (nexmifa mutant) to characterize PMN morphology. The selected sgRNA-Cas9 system effectively inserted a 159 bp frameshift mutation that prematurely altered protein translation and produced a truncated protein (Figure 2). There was no obvious difference in appearance between the two groups of zebrafish in the bright field (Supplementary Figure S1). In order to better understand the morphological changes of motor neurons, firstly, we drew a schematic for three different PMNs in one hemisegment in the spinal cord (Figure 3A). Secondly, we observed abnormal PMNs at 48 and 72 hpf under fluorescence microscope. We found the abnormalities in nexmifa mutant included the loss of Cap and/or Mip, motor neuron loss, reduced Cap length, and abnormal Cap branches (Figure 3B). Statistical analyses revealed the percentage of embryos with normal PMNs was lower than controls (55.5% ± 3.7% vs. 97% ± 1.4%) at 48 hpf and 57.5% ± 5.1% vs. 96% ± 1.9% at 72 hpf (Figure 3C). Cap development was also restricted, e.g., the Cap length in axons in nexmifa mutants was shorter than controls (103.8 μm ± 29.3 μm vs. 173.5 μm ± 10.6 μm) at 48 hpf. When embryos developed to 72 hpf, mutant Cap lengths had grown, however, they remained shorter than controls (130.8 μm ± 28.8 μm vs. 203.8 μm ± 13.7 μm) (Figure 3D). These observations indicated that truncated axons had not completely recovered. In addition, Cap branches were also abnormal between controls and mutants; branch numbers were significantly lower in mutants than controls (47 ± 11 vs. 160 ± 17) at 48 hpf, and at 72 hpf, branches were less than controls (95 ± 21 vs. 175 ± 19) and were more disordered (Figure 3E).

To specifically confirm that motor neuron defects were caused by nexmifa loss, we established a knockdown nexmifa fish model by injecting a splice-blocking MO into one-cell stage zebrafish embryos. At 72 hpf, post-nexmifa-MO injection, splice-blocking MO effects were checked and quantitated by RT-PCR, then confirmed by sequencing. MO-nexmifa generated a larger alternatively spliced RT-PCR product [452 bp vs. 271 bp (Control MO)] (Supplementary Figure S2B). After sequencing, we confirmed that nexmifa-MO injection had caused intron 2 (181 bp) to be retained in nexmifa mRNA (Supplementary Figure S2C), resulting in a reading frame shift to generate successful nexmifa knockdown. We also investigated PMN morphology in nexmifa-MO fish at 48 and 72 hpf; the results were similar to those in nexmifa mutants. We also performed rescue experiments by co-injecting nexmifa mRNA with nexmifa-MO to confirm phenotypic changes induced by nexmifa-MO injection. We observed that this strategy partly rescued abnormal motor neuron (Supplementary Figure S3A). For example, the percentage of normal embryos was recovered from 62.3% ± 3.5% to 75.5% ± 4.0% at 48 hpf and 60.5% ± 6.0% to 73.4% ± 5.8% at 72 hpf (Supplementary Figure S3B). Also Cap length was recovered from 101.5 μm ± 16.2 μm to 133.2 μm ± 21.5 μm at 48 hpf and from 126.1 μm ± 34.4 μm to 175.6 μm ± 25.6 μm at 72 hpf (Supplementary Figure S3C). The number of Cap branches was also recovered from 57 ± 12 vs. to 126 ± 29 at 48 hpf and from 88 ± 21 to 119 ± 34 at 72 hpf, and were less disordered than the nexmifa morphant group (Supplementary Figure S3D).

### Nexmifa Knockout Mutants Display Impaired Motility

**FIGURE 4:**
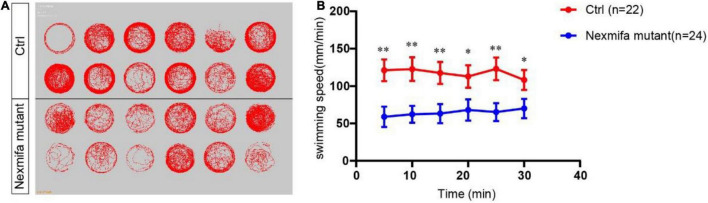
Motor defects in nexmifa mutant zebrafish embryos at 7 dpf. **(A)** Thirty minutes of free movement trajectories of control and nexmifa mutants. **(B)** Quantification of swimming distances of control and nexmifa mutants at 7 dpf per 5 min (*n* = 22 and 24, respectively). Each point represents the mean ± standard error of the mean (SEM). *p < 0.05 and ^**^p < 0.01.

To investigate if motor neuron defects affected motor ability, video-tracked swimming activities were performed for 30 min at 7 dpf. As shown (Figure 4), movement trajectories in nexmifa mutants were significantly decreased when compared with controls (Figure 4A). The swimming distance per 5 min decreased in nexmifa mutants when compared with controls (Figure 4B), consistent with movement trajectories.

### Transcriptomic Profiling of Nexmifa Morphants and Control Zebrafish

**FIGURE 5:**
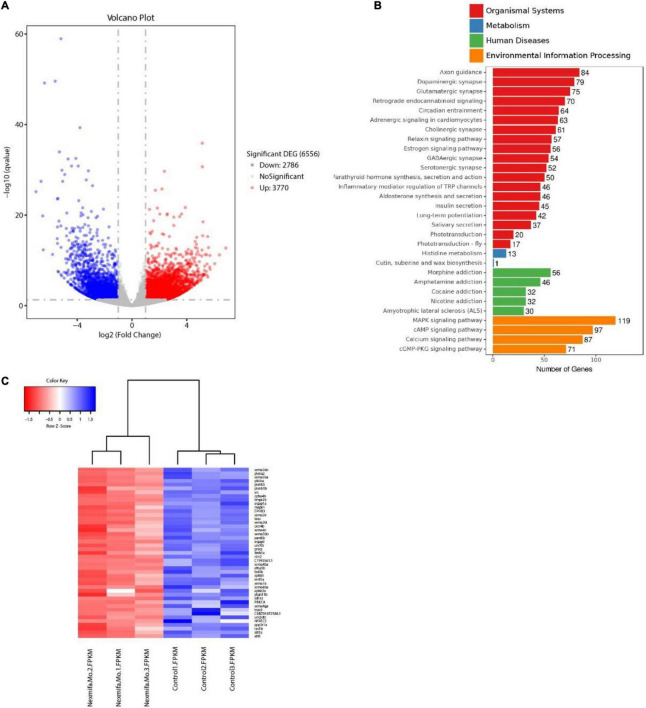
Transcriptomics profiling in nexmifa morphant and wild-type zebrafish. **(A)** Volcano map showing DEGs in control and nexmifa morphants. Red and blue spots represent up-regulated and down-regulated genes, respectively. **(B)** KEGG pathway annotation of DEGs in control and nexmifa morphants. **(C)** Heatmap pathway differences between control and nexmifa morphants showing down-regulated genes in axon guidance.

**FIGURE 6:**
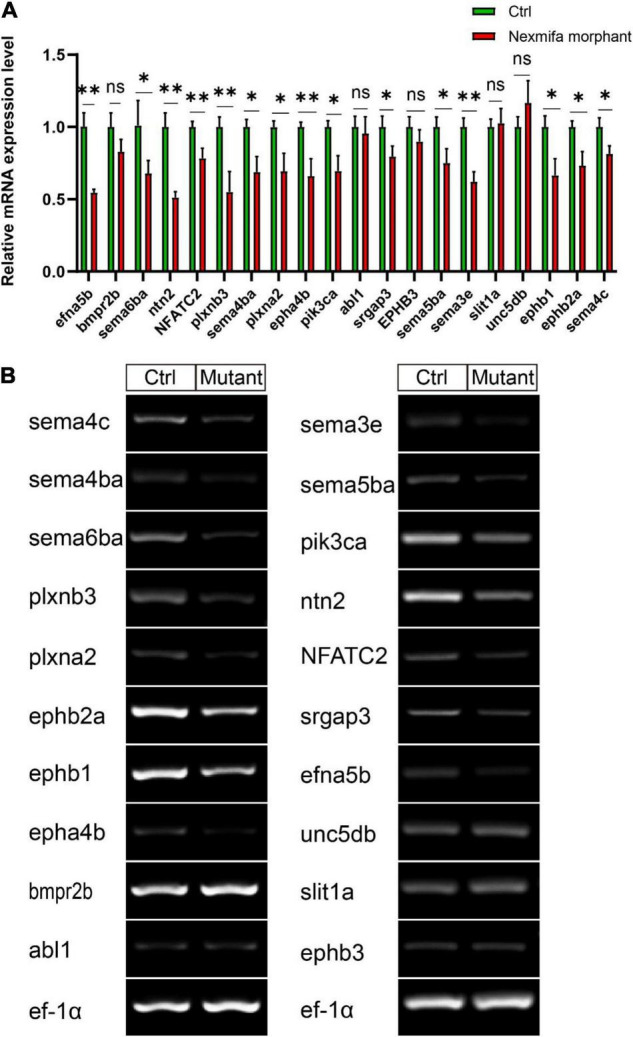
The expression of 20 down-regulated DEGs. **(A)** Expression of 20 down-regulated DEGs between Ctrl and nexmifa morphant by qRT-PCR. Bars represent the mean ± standard deviation (SD). *p < 0.05 and ^**^p < 0.01. ns: non-significant. **(B)** Expression of 20 down-regulated DEGs between Ctrl and nexmifa mutant by RT-PCR.

To identify mechanisms where nexmifa may have affected motor neuron morphogenesis, we performed RNA sequencing using RNA samples from control and nexmifa morphant zebrafish at 72 hpf. We identified 6,556 differentially expressed genes (DEGs), with 3,770 up-regulated and 2,786 down-regulated DEGs between the two groups (fold change > 2 or < 0.5, *p* < 0.05) (Figure 5A and Supplementary Table S2). According to Kyoto Encyclopedia of Genes and Genomes (KEGG) annotations, many DEGs were involved in axon guidance pathways and various synaptic pathways. In particular, and according to Organismal Systems, DEG numbers involved in axon guidance (84) were the highest when compared with other groups (Figure 5B). We also observed 45 down-regulated DEGs in the axon guidance pathway (Figure 5C). Among these 45 DEGs, the top 3 genes with the largest fold changes are efna5b, bmpr2b and sema6ba. To verify the reliability of RNA-seq, we not only further test the expression of efna5b, bmpr2b and sema6ba but also test other 17 down-regulated DEGs randomly by qRT-PCR at 72 hpf. Then we found the expression of the most genes including efna5b and sema6ba were consistent with the RNA-seq results (Figure 6A). Moreover, we test the expression of the above 20 genes between Ctrl and nexmifa mutant by RT-PCR and gained the similar trend change (Figure 6B).

### Efna5b and Sema6ba Overexpression Rescues the Motor Neuron Defects and Impaired Motility in Nexmifa Mutant Embryos

**FIGURE 7:**
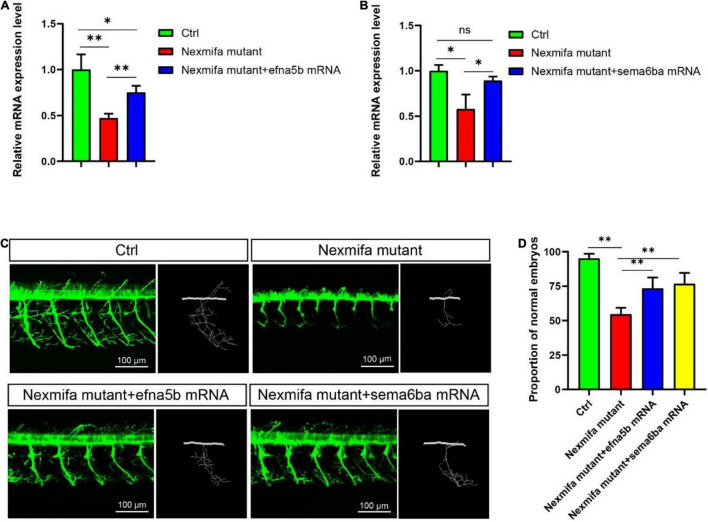
Efna5b and sema6ba overexpression rescues motor neuron defects in nexmifa morphant embryos. **(A)** Expression of efna5b after injection of efna5b mRNA in nexmifa mutant embryos at 72 hpf. **(B)** Expression of sema6ba after injection of sema6ba mRNA in nexmifa mutant embryos at 72 hpf**. (C)** Abnormal PMNs in nexmifa morphant zebrafish were rescued by injecting efna5b or sema6ba mRNA. **(D)** Percentage of zebrafish embryos with normal Cap primary motor neurons (*n* = 150, 242, 250, and 257, respectively). *p < 0.05 and ^**^p < 0.01. ns: non-significant.

**FIGURE 8:**
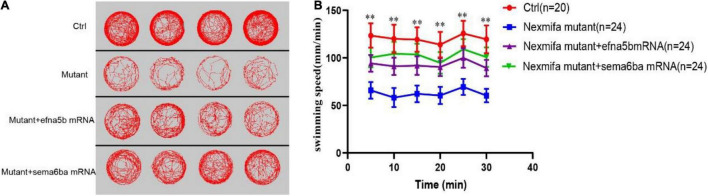
Efna5b and sema6ba overexpression rescues the impaired motility in nexmifa mutant embryos at 7 dpf. **(A)** Thirty minutes of free movement trajectories among control, nexmifa mutants, nexmifa mutant + efna5b mRNA and nexmifa mutant + sema6ba mRNA. **(B)** Quantification of swimming distances among control, nexmifa mutants, nexmifa mutant + efna5b mRNA and nexmifa mutant + sema6ba mRNA at 7 dpf per 5 min (*n* = 20, 24, and 24, respectively). Each point represents the mean ± standard error of the mean (SEM). ^**^p < 0.01.

As the downregulation of efna5b and sema6ba in nexmifa loss of function embryos, we hypothesized if nexmifa regulated motor neurons in zebrafish by down-regulating efna5b and sema6ba expression. To confirm this, we synthesized efna5b and sema6ba mRNA *in vitro*, and injected molecules into the yolk of a one-cell stage nexmifa mutant embryos. Then, we found the relative mRNA expression of efna5b and sema6ba were significantly up-regulated compared with nexmifa mutants by qRT-PCR, which indicate the successful of overexpression (Figures 7A–B). We observed nexmifa mutant embryos had significantly reduced motor neuronal defects caused by nexmifa loss (Figure 7C). Only 54.5% ± 4.6% of embryos presented normal PMNs in nexmifa mutants at 48 hpf, whereas this percentage increased to 73.3% ± 7.9% after efna5b mRNA injection and 76.7% ± 7.9% after sema6ba mRNA injection (Figure 7D). As shown (Figure 8A), the movement trajectory was dramatically increased when the mutants were injected with efna5b RNA or sema6ba RNA compared with that in the nexmifa mutant at 7 dpf. Consistent with movement trajectory, the swimming distances per 5 min was also dramatically increased when the mutants were injected with efna5b RNA or sema6ba RNA compared with that in the nexmifa mutant (Figure 8B). The results demonstrated that efna5b and sema6ba overexpression could rescue the motor neuron defects and impaired motility which caused by loss of nexmifa.

## Discussion

Previous studies demonstrated that nexmif was involved in neurite morphological development, cell to cell and cell to matrix adhesion, cell migration, and maintained normal synaptic formation in neurons (Ishikawa et al., 2012; Magome et al., 2013; Gilbert et al., 2020), however, little was known about nexmif function in spinal motor neuron development. Additionally, most studies have explored the *in vitro* effects of nexmif on neurite morphology (Van Maldergem et al., 2013), but none have done this *in vivo*. Here, our *in vivo* nexmifa expression and deficiency phenotype data provided new insights on nexmifa functions in regulating the morphogenesis of spinal motor neuron in zebrafish.

In mouse brains, nexmif mRNA expression commences as early as E10.5, increases throughout development and peaking at P3, but continues at lower levels into adulthood (Cantagrel et al., 2009; Ishikawa et al., 2012). Our WISH data showed that almost all nexmifa was expressed in the brain and spinal cord; expression was observed from 20 hpf, whereas at 48 hpf, expression peaked and then gradually decreased. These spatiotemporal expression patterns in zebrafish were similar to mice. Furthermore, using flow cytometry, we sorted motor neurons from the Tg(mnx1:GFP)ml2 transgenic zebrafish line as these motor neurons were labeled by GFP. RT-PCR data showed that nexmifa was highly expressed in GFP-positive cells, indicating that nexmifa may directly regulate motor neuron development in the spinal cord.

Embryo and larva motor neurons are similar in morphology and projection patterns with respect to adult motor neurons. All primary motoneurons are born between 9 and 16 hpf. During PMN development, these cells extend their axons along stereotyped pathways and develop branches to invade into the myotome to form distributed neuromuscular synapses to nerve musculature. At 48 hpf, the Cap somata are located within a short distance of the ventral root, with axons following a stereotyped pathway down the middle of the segment, making a collateral or varicosity at the horizontal septum. At the ventral edge of the musculature, each axon turns dorsally and laterally grows along the rostral myoseptum (Myers et al., 1986). At 72 hpf, exuberant branches are formed and further invade into the myotome to form distributed neuromuscular synapses (Liu and Westerfield, 1990; Downes and Granato, 2004). To explore if nexmifa was involved in the morphogenesis of motor neurons in the spinal cord, we established knockout and knockdown fish models. Our data showed that both models exhibited obvious motor neuron loss and defects in PMN axons. Moreover, after coinjecting nexmifa mRNA with nexmifa-MO, truncated Cap and disordered branches were partly rescued. Thus, nexmifa helped regulate axon morphology.

Motoneurons establish important connections between the CNS and muscle. If they develop incorrectly, they cannot form the required connections, resulting in movement defects or paralysis (Hao le et al., 2017). Previous studies showed several motor defects were related to abnormal PMN development in zebrafish (Brusegan et al., 2012; Gong et al., 2017). In our study, impaired motility was consistent with the motor neuron defects seen in nexmifa knockout zebrafish.

Previous studies also showed that when motor neuron dendrites are reduced, motoneurons receive less innervation, leading to decreased activity (Gao et al., 2020; Zhu et al., 2021). As swimming involves alternating side-muscle contractions caused by alternating motor neuron activation, less active motor neurons could lead to a reduction in alternating muscle contractions and less distance moved (Hao le et al., 2017). Thus, we hypothesized this impaired motility was due to a decreased number of branches induced by nexmifa loss. Both musculature and motor neuron are responsible for embryonic motility (Menelaou et al., 2008), however, whether nexmifa affects muscle development warrants further study.

Many genes are involved in motor neuron development via morphogenesis regulation (Dong et al., 2019; Koh et al., 2020). In this study, RNA-sequencing was performed on control and nexmifa morphant embryos to explore nexmifa-mediated morphogenesis mechanisms. Several DEGs were related to CNS development, e.g., DEGs were involved in the axon guidance pathway and various synapse pathways, consistent with mouse data (Ishikawa et al., 2012; Gilbert et al., 2020). This consistency not only indicated successful model establishment (knockdown), but also demonstrated the conserved function of nexmif. Of the 45 down regulated DEGs related to axon guidance, efna5b, bmpr2b and sema6ba are the top three genes with the largest fold changes. Using qRT-PCR at 72 hpf, we found that expression of most DEGs including efna5b and sema6ba were consistent with RNA-sequencing results, thereby proving RNA-sequencing data reliability. Efna5b, or ephrin-A5b, belongs to the family of epha, Eph receptor tyrosine kinases and their cognate ligands. Ephrins are a important class of axon guidance molecules (Lisabeth et al., 2013; Cayuso et al., 2015). EphrinA6 drastically reduces BDNF-induced axon branching (Poopalasundaram et al., 2011), whereas *Caenorhabditis elegans* ephrin EFN-4 promotes primary neurite outgrowth in AIY interneurons and D-class motor neurons (Schwieterman et al., 2016). Sema6ba belongs to the semaphorins (Semas), another large class of proteins that function throughout the nervous system to guide axons. In Sema-2b loss-of-function embryos, specific motor neuron and interneuron axon pathways display guidance defects (Emerson et al., 2013). Sema5A was also expressed in the myotome during the period of motor axon outgrowth, the lack of sema5A in zebrafish result in delayed in motor axon extension into the ventral myotome and aberrant branching of these motor axons (Hilario et al., 2009). We showed that nexmifa deficiency caused a significant decrease in efna5b and sema6ba expression levels. Furthermore, efna5b and sema6ba overexpression rescued the motor neuron defects and inactive swimming behavior in nexmifa mutant embryos. These data suggested that nexmifa regulated motor neuron development, at least in part, by regulating efna5b and sema6ba expression. In the future, we will perform dual-luciferase reporter gene assays to confirm interactions between nexmifa and efna5b and sema6ba.

## Data Availability Statement

The datasets presented in this study can be found in online repositories. The names of the repository/repositories and accession number(s) can be found below: NCBI BioProject PRJNA797475.

## Ethics Statement

The animal study was reviewed and approved by Administration Committee of Experimental Animals, Jiangsu Province, China.

## Author Contributions

HN and Y-JW designed the study. Y-QZ and G-HS performed the experiments. DL and H-YL analyzed the data and are responsible for the statistical analysis. Y-QZ wrote the manuscript. All the authors have reviewed and approved this version of the manuscript.

## Conflict of Interest

The authors declare that the research was conducted in the absence of any commercial or financial relationships that could be construed as a potential conflict of interest.

## Publisher’s Note

All claims expressed in this article are solely those of the authors and do not necessarily represent those of their affiliated organizations, or those of the publisher, the editors and the reviewers. Any product that may be evaluated in this article, or claim that may be made by its manufacturer, is not guaranteed or endorsed by the publisher.

## Supplementary Material

Supplementary Figure S1 Appearance of embryo fish at 72 hpf between Ctrl and nexmifa mutant.

Supplementary Figure S2 The effect of MO on knocking down nexmifa expression. **(A)** An nexmifa splice-blocking MO was designed to target the exon 2-intron 2 splice donor site. **(B)** RT-PCR analysis of total RNA from 24 hpf embryos treated with or without MO nexmifa (Std MO). **(C)** Schematic and sequencing data showing that 181 bp of intron 2 were added between exons 2 and 3.

Supplementary Figure S3 Nexmifa affects motor neuron morphogenesis in nexmifa morphant zebrafish embryos. **(A)** Confocal imaging of primary motor neurons in three different groups at 48 and 72 hpf. **(B)** The percentage of embryos with normal PMNs in the three groups at 48 hpf (*n* = 113, 231, and 218) and 72 hpf (*n* = 104, 214, and 221, respectively). **(C)** Cap axon lengths in the three groups at 48 hpf (*n* = 18, 29, and 21, respectively) and 72 hpf (*n* = 17, 23, and 25, respectively). **(D)** The number of branches per 1 mm Cap axons in the three groups at 48 hpf (*n* = 7, 9, and 10, respectively) and 72 hpf (*n* = 8, 10, and 10, respectively). Bars represent the mean ± standard deviation (SD). ^**^*p* < 0.01.

Supplementary Table S1 Primers and oligos are used in this study.

Supplementary Table S2 DEGs between Ctrl and morphants.
