# Supplementary figures and images for: Nexmifa Regulates Axon Morphogenesis in Motor Neurons in Zebrafish
Source: Front Mol Neurosci. 2022 Mar 31;15:848257. doi: 10.3389/fnmol.2022.848257 (PMC9009263; doi:10.3389/fnmol.2022.848257)

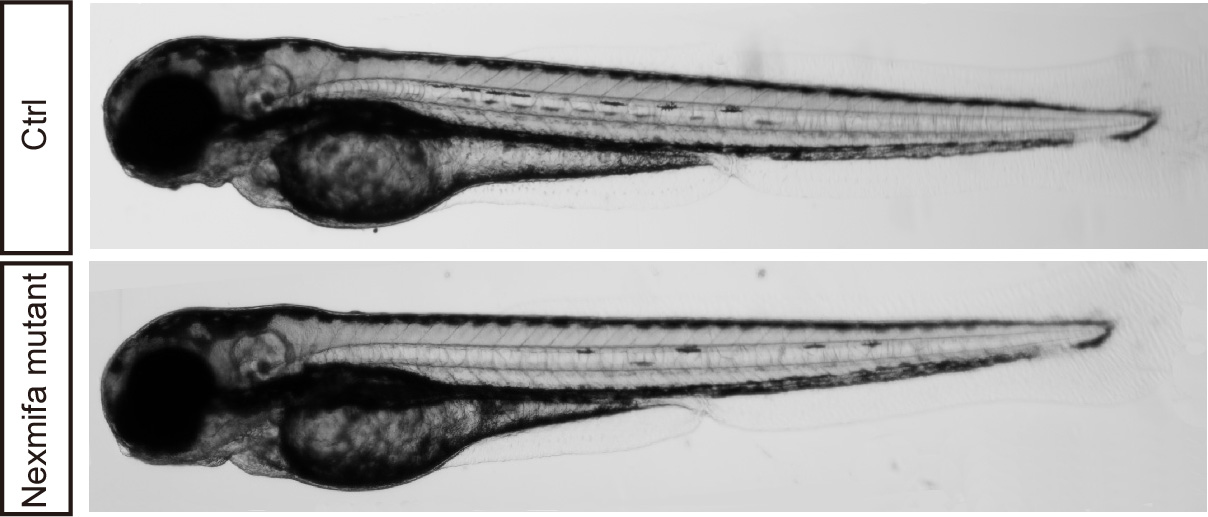

Supplement: Supplementary Figure S1 — Appearance of embryo fish at 72 hpf between Ctrl and nexmifa mutant. [file Image_1.JPEG]

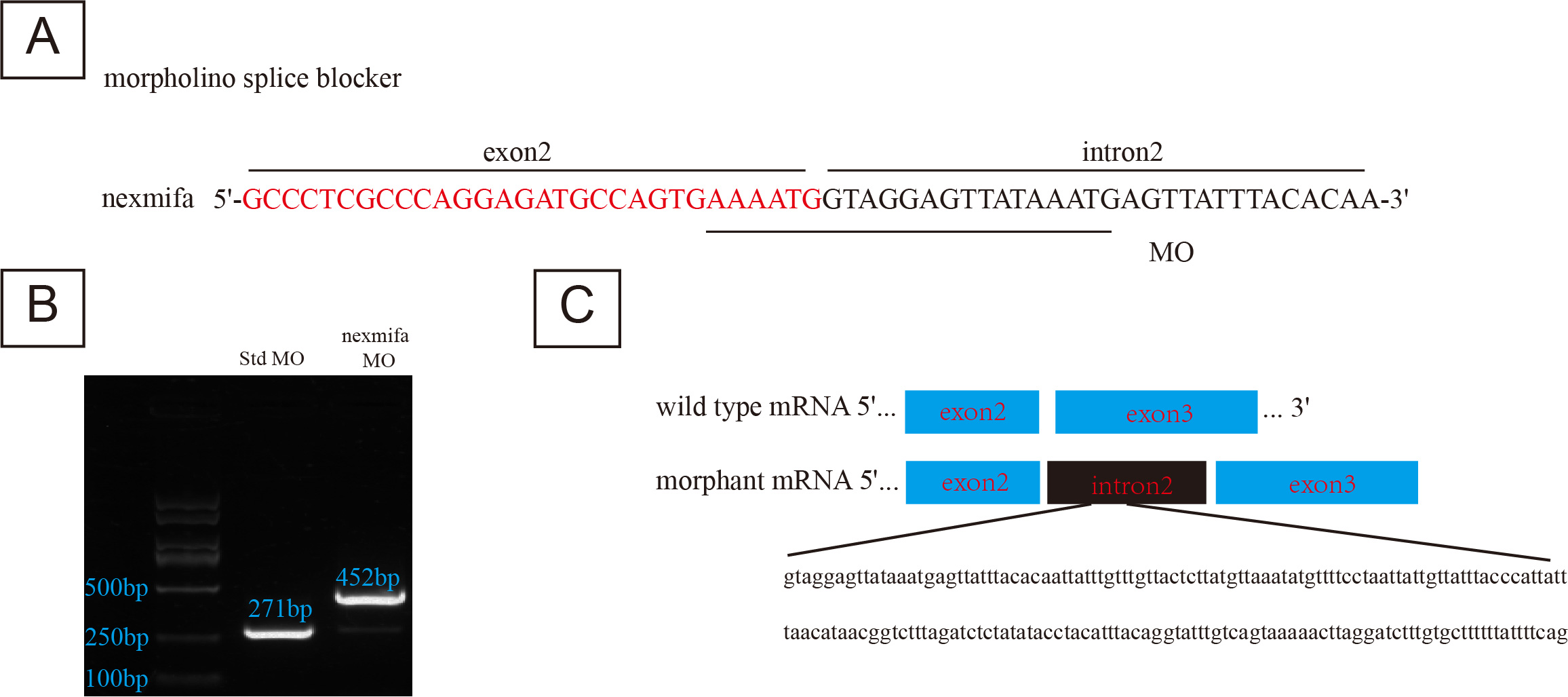

Supplement: Supplementary Figure S2 — The effect of MO on knocking down nexmifa expression. (A) An nexmifa splice-blocking MO was designed to target the exon 2-intron 2 splice donor site. (B) RT-PCR analysis of total RNA from 24 hpf embryos treated with or without MO nexmifa (Std MO). (C) Schematic and sequencing data showing that 181 bp of intron 2 were added between exons 2 and 3. [file Image_2.JPEG]

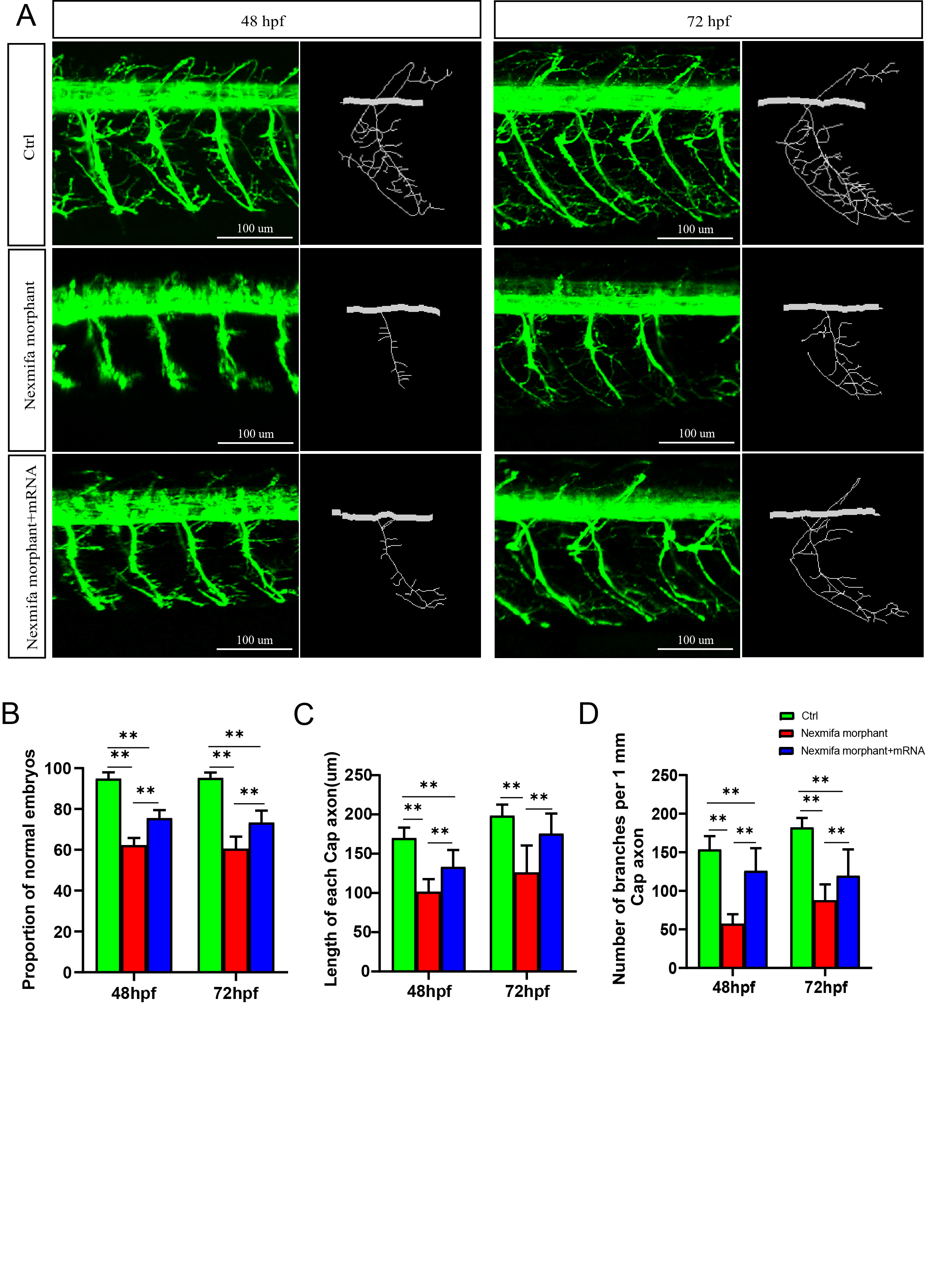

Supplement: Supplementary Figure S3 — Nexmifa affects motor neuron morphogenesis in nexmifa morphant zebrafish embryos. (A) Confocal imaging of primary motor neurons in three different groups at 48 and 72 hpf. (B) The percentage of embryos with normal PMNs in the three groups at 48 hpf (n = 113, 231, and 218) and 72 hpf (n = 104, 214, and 221, respectively). (C) Cap axon lengths in the three groups at 48 hpf (n = 18, 29, and 21, respectively) and 72 hpf (n = 17, 23, and 25, respectively). (D) The number of branches per 1 mm Cap axons in the three groups at 48 hpf (n = 7, 9, and 10, respectively) and 72 hpf (n = 8, 10, and 10, respectively). Bars represent the mean ± standard deviation (SD). **p < 0.01. [file Image_3.JPEG]
