# Supplementary material for: Nexmifa Regulates Axon Morphogenesis in Motor Neurons in Zebrafish
Source: Front Mol Neurosci. 2022 Mar 31;15:848257. doi: 10.3389/fnmol.2022.848257 (PMC9009263; doi:10.3389/fnmol.2022.848257)
Supplement: Supplementary Table S1 — Primers and oligos are used in this study. [file Table_1.DOCX]

| **Supplementary Table 1 Primers used in this study.** | |
| --- | --- |
| **Primer name** | **Sequence** (5’-3’) |
| nexmifa -probe-F | GGGGATGGTACGAATGTTGC |
| nexmifa -probe-R | GCTGTGGGGAAGAGTTAGGT |
| nexmifa-test-F | AGATCACGGGCTCTCCCTCC |
| nexmifa-test-R | CTTGCCTGCCAGTATGCTTTTA |
| nexmifa - sgRNA -R | AAAAAAAGCACCGACTCGGTGCCAC |
| nexmifa -sgRNA-F | TAATACGACTCACTATAggtgcccgaagaagaggcggcggGTTTTAGAGCTAGAAATAGC |
| ef1α-F | CTTCAACGCTCAGGTCATCA |
| ef1α-R | CTTCAACGCTCAGGTCATCA |
| efna5b-F | GCAGGCGGAGATGATCGTGTTC |
| efna5b-R | TTCGGTTCCAGAAGACAGCATATCG |
| sema6ba-F | GGATGAGCCCGAAAGCCTTGTC |
| sema6ba-R | GACGACGGAGTGTTGCTGTGAG |
| ntn2-F | CCGCTTTCTGGAGGTCTCATTGC |
| ntn2-R | GACGACTGAACACCACCCGAATATC |
| NFATC2-F | TGTGAGCAGGATATGAAGGGCAAAC |
| NFATC2-R | TTCCAGAGACGGCAGAGACGAC |
| plxnb3-F | GGAGGAGTGCTGGCTGTAGAGG |
| plxnb3-R | AGAGGCTGGACATCAGGTGGTTC |
| plxna2-F | CCGAGAGGAAGTTTCAGTGTGGATG |
| plxna2-R | CATTGGTGCTGGCGAGGTGTAG |
| epha4b-F | GTGCTGGCGACGGAAGGATATG |
| epha4b-R | ACGCTGAAGGTGTAGTTGGTGTTG |
| pik3ca-F | TGCCAACCTCTTCATCAACCTCTTC |
| pik3ca-R | AGTCTTGCGGATGTAAGCGATGTC |
| abl1-F | AGTTAGTGGGTGAGCAGAGGAGAC |
| abl1-R | ACTCGTGGACCGTGAACACATTG |
| srgap3-F | GCTTTGCCGCTGAGATTGTAATGC |
| srgap3-R | TTCTGTGCCCATCCCGTTTATGTG |
| EPHB3-F | CATTCCTCGCAACTGACACTCCTC |
| EPHB3-R | TGTGGCATCATCCTCCTCATCCTC |
| sema5ba-F | GGTCTGCTGTCTTTCCTGGTGTATG |
| sema5ba-R | TCTTTGGTGTGGTGTTGCCCTTG |
| sema3e-F | AGGAGCGAGAGTTCAGGTTGGAG |
| sema3e-R | TTGATGCTAAAGGGCTGTTGGGATG |
| slit1a-F | AACAACAACAACATCAGCAGCATCC |
| slit1a-R | CCACGACAGCACACAGTCACAG |
| unc5db-F | TGCTGATGACACCTCTTGGGAAATG |
| unc5db-R | ACAGGACAGGACAGGCTTAGGC |
| ephb1-F | GTAACCTGCTGGCTCACACACTC |
| ephb1-R | GCCTGGTTGGTGGTGATGTCTATG |
| ephb2a-F | TAATACGGCTTGTGTGGGCTGTTG |
| ephb2a-R | TCTCGTCGTATCCACTCACTTCCTC |
| sema4c-F | GGGCGTGAAGTTCCAGACTGAAAG |
| sema4c-R | AGCAACAGACAAGCAGCACCTAAG |
| bmpr2b-F | CAGCAGACGACGATGGCAGTAAG |
| bmpr2b-R | GCGAGTTCACAGGCAGGTCATATC |
| sema4ba-F | GCCTCTGCTGTTGAAACGAAATGTG |
| sema4ba-R | TTGTGGATTCTGCCGTCATCTGTG |
| nexmifa-mRNA-BamH1-F | CGGGATCCCGACCAAACGGAGGCTTAAG |
| nexmifa-mRNA-Xho1-R | CCGCTCGAGGAACGGAGCAAAAGAAGGGG |
| nexmifb-mRNA-Cla1-F | CCATCGATACTGATGTGTCCCCAAACCA |
| nexmifb-mRNA-Xho1-R | CCGCTCGAGAGAAGGTGGGGATTCTAAAGCA |
| efna5b-mRNA- BamH1-F | CGGGATCCCCAGCCTCCATGATCACAGA |
| efna5b-mRNA-EcoR1-R | CGGAATTCCCGGCTTTGATGGTGTGATGT |
| sema6ba-mRNA- EcoR1-F | CGGAATTCCGCATCACCTTGAGGATTTT |
| sema6ba-mRNA- Xba1-R | GCTCTAGAATGAGGTTTCATCCGCTCAC |
